# A comparative study of chromatic dispersion compensation in 10 Gbps SMF and 40 Gbps OTDM systems using a cascaded Gaussian linear apodized chirped fibre Bragg grating design

**DOI:** 10.1016/j.heliyon.2022.e09308

**Published:** 2022-04-22

**Authors:** Isidore Nsengiyumva, Elijah Mwangi, George Kamucha

**Affiliations:** a Department of Electrical Engineering, Pan African University, P.O. Box 62000, Nairobi, 00200, Kenya; b Faculty of Engineering, University of Nairobi, P.O. Box 30197, Nairobi, 00100, Kenya

**Keywords:** Chromatic dispersion compensation, Apodized chirped fibre Bragg grating, Optisystem

## Abstract

Optical time domain multiplexed (OTDM) technology helps to respond to the need for increased capacity and demand in internet traffic using optical transmissions. High-speed optical networks are highly affected by chromatic dispersion (CD) that causes pulse broadening and intersymbol interference (ISI) at the light detector. This paper presents a comparative analysis of chromatic dispersion compensation on 10 Gbps single mode fibre (SMF) and 40 Gbps OTDM operating at C-band using Gaussian apodized linear chirped fibre Bragg grating, Erbium Doped Fibre Amplifier (EDFA) and uniform fibre Bragg grating (UFBG) hybrid approach in OptiSystem 18. A cascade of four UFBG and chirped Bragg gratings design approach presents minimal improvement in the quality factor and achievable single mode fibre length. Quality factor results at a reference wavelength of 1550 nm were obtained for respective chirped Bragg grating lengths of 8 mm, 20 mm, 50 mm, 87 mm, and 90 mm using linear Gaussian apodization. The Q-factor results obtained using the proposed simulation model for single 10 Gb/s channel were respectively 51.14 dBm over 30 km at a grating length of 20 mm and 15.64 dBm over 150 km at 45 mm of grating length. Different eye-diagrams corresponding to varying grating lengths have also been presented for 10 Gb/s and 40 Gb/s at corresponding SMF lengths.

## 1 Introduction

Laser sources are generally thin but not monochromatic. The optical pulses inside a fibre propagate at different speeds and thus leading to relative delays in arrival times. This effect is termed chromatic dispersion and is statistical in nature and predominant form of dispersion in the fibre [1]. It causes intersymbol interference (ISI) due to the intermixing of slower and faster wavelengths. As light travels through the optical fibre, impairments due to the quality of the injected signal, nature and design of the fibre itself and the distance covered have adverse effects on the signal. An optical fibre has dispersion which is in the range of 15–20 ps/nm/km and becomes more profound at long distances and higher transmission rates [2, 3]. Many laser source designs have been proposed by researchers especially the short-pulsed Raman fibre lasers (RFL). The RFL is credited with considerable wavelength flexibility and broadband tuning range. However, ultrafast RFLs still have the main challenge of successfully generating temporally stable femtosecond pulses with a high SNR [4].

In order to deal with the problems caused by dispersion in a fibre, solutions proposed by researchers over the years have incorporated the design of low dispersion fibres, dispersion compensating or shifted fibres, use of fibre Bragg gratings (FBG) and the digital regeneration of the signal [5, 6, 7, 8]. Dispersion compensation fibre (DCF) and Fibre Braggs Grating (FBG) are the most widely used elements to compensate various types of dispersion effects links [9]. The DCF element has negative dispersion and presents non-linear effects in long distance optical communication. However, the performance reduces with the increase in distance and transmission rate when different non-linearities are considered in system design, and many other external factors in the field of installation like temperature, stress, and external load [10]. The FBG technique deals successfully with the limitations of dispersion compensated fibres and have low-cost and minimum non-linear effects [2]. The chirped FBG allows the variation of the period of the fibre grating using linear, quadratic, or cubic trends. The linearly chirped grating has found a special place in optics as a dispersion-correcting and compensating device [11]. Moreover, designs of chirped FBG have been proposed to generate efficient and optimized high power femtosecond pulses in mode-locked fibre lasers [12]. The chirped FBG have also been used in the study and experimental observation of optical rogue waves (also known as “freak waves”) [13]. Real-time measurement of optical waves is an important phenomenon that can help to explain the behavior and properties of rogue waves and their prospects in improving the quality of non-linear optical fibre communications.

Optical fibre technology has helped to deal with the explosive increase in internet demand and low-cost transmissions. However, it suffers from several effects such as attenuation and dispersion. Different types of amplifiers such as Raman amplifier, and the Erbium doped fibre amplifier (EDFA) have been employed to mitigate attenuation effects [14]. The works presented in [8] uses a combination of EDFA and FBG to reduce dispersion effects on both a single channel and four-channel system. In [15], chromatic dispersion is compensated using chirped FBG in conjunction with EDFA to reduce attenuation for wave division multiplexed (WDM) optical system using four channels. Mohammed et al. [16] used a *tanh* function apodized chirped fibre jointly with dispersion compensated fibre (DCF) and obtained significant results using a linear apodization. A fibre Bragg grating technique using linear chirp was used in [17] with good results and its performance compared against various other dispersion compensation techniques like the DCF, and the joint DCF-FBG approach. Meena et al. [18] presented a design of dense WDM network with linear chirped fibre Bragg grating and evaluated its performance in terms of bit error rate (BER) and Q-factor. In [18], Q-factor results were obtained for different SMF lengths using a core refractive index of 1.47: for an SMF length of 150 km for example, corresponding BER and Q-factor results were found to be respectively 1.63×10^−37^ and 12.74 dBm. In [19], A. F. Sayed et al. presented a cascaded UFBG design to enhance WDM optical transmissions and used *Optisystem 7* to simulate results for different fibre lengths, input power and FBG length. Results for four cascaded uniform FBG design on the transmitter part yield better reflectivity than simply using one, two or three uniform FBG in cascade. This paper relies on simulations in *Optisystem 18* software to design and simulate our proposed model since it is costly and difficult to experiment in real-life environment.

The rest of this paper is organized as follows: Section 2 presents the mathematical analysis of uniform and chirped fibre Bragg grating. Section 3 gives the experimental setup for 10 Gb/s and 40 Gb/s single channel transmissions, and the important parameters used in simulation. A discussion of the results is presented in Section 4 while Section 5 presents a conclusion of the research investigation.

## 2 Mathematical analysis of uniform and chirped fibre Bragg grating

**Figure 1:**
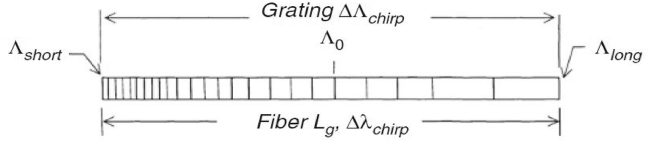
Chirped fibre grating schematic.

In this section, equations for uniform and chirped fibre Bragg grating, optical signal-to-noise ratio (OSNR), Q-factor and the bit error rate (BER) and other important parameters are presented. Figure 1 illustrates a schematic of a chirped grating, of length L_g_, grating period Λ_0_ and chirped bandwidth $\Delta \lambda_{chirp}$.

The variation of the chirp is given by the difference between the short and long wavelengths is given in Eq. (1) [11]:

(1) $$\Delta \lambda_{chirp}=2n_{eff}\left(\Lambda_{long}-\Lambda_{short}\right)=2n_{eff}\Delta \Lambda \;$$

where *n*_*eff*_ is the effective refractive index.

The dispersion of the chirped FBG, $D_{g}$, is related to the $n_{eff},$ length l of the fibre grating and chirp variation $\Delta \Lambda_{chirp}$ and is given by Eq. (2).

(2) $$D_{g}=\frac{2n_{eff}}{\Delta \lambda_{chirp}c}l$$

where *c* is the speed of light.

Light entering a chirped FBG suffers a delay *τ* on reflection and experiences dispersion due to a maximum delay of $\frac{2L_{g}}{v_{g}}$ between the shortest and longest wavelengths derived in [11] and given in Eq. (3).

(3) $$\tau \left(\lambda \right)=\frac{\left(\Lambda_{0}-\Lambda \right)}{\Delta \lambda_{chirp}c}\frac{2L_{g}}{v_{g}}=\frac{D_{g}\left(\Lambda_{0}-\Lambda \right)}{2n_{eff}l}\frac{2L_{g}}{v_{g}}\;\text{for}2n_{eff}\Lambda_{short}<\lambda <2n_{eff}\Lambda_{long}$$

where $\Lambda_{0}$ is the Bragg wavelength at the center of the chirped bandwidth of the grating and $v_{g}$ is the group velocity of light in the fibre.

The overall chromatic dispersion, $D_{ch}$, along a fibre length *L*, is derived in [20] and is related to the delay in the fibre as given in Eq. (4):

(4) $$\frac{\Delta \tau }{L}=D_{ch}\left(\Delta \Lambda \right)$$

where (ΔΛ) is the finite width of the source spectrum.

The Bragg wavelength along the core of the chirped fibre grating $\Lambda_{B}\left(z\right)$ when a frame of reference along the +z direction is considered and with a refractive index, *n*(*z*), is given by Eq. (4) as in [19]:

(5) $$\Lambda_{B}\left(z\right)=2n\left(z\right)\Lambda \left(z\right)$$

A linear variation from a period $\Lambda_{0}$ to Λ(z) is given by:

(6) $$\Lambda \left(z\right)=\Lambda_{0}+xz$$

The refractive index n(z) i s also a function of the effective index $n_{eff}$ of the fibre, the average change in the index modulation Δn and the apodization function g(z) as expressed in [19] by Eq. (7):

(7) $$n\left(z\right)=n_{eff}+\Delta ng\left(z\right)cos(\frac{2\pi z}{\Lambda_{0}}\left(1+xz\right))$$

Ultimately, the optical to signal noise ratio (OSNR), the quality factor and the bit-error rate (BER) are the most important parameters that characterize the performance of the transmission link as derived from the eye-diagrams. Considering an input power $P_{in}$, total dispersion $D_{t}$, an attenuation coefficient α and dispersion coefficient $D_{f}$ of the optical fibre, amplifier noise figure $N_{f}$, a frequency υ and a bandwidth frequency $\Delta \upsilon_{0}$ , the OSNR is given by [21]:

(8) $$OSNR=\frac{P_{in}D_{f}}{N_{f}h\upsilon \Delta \upsilon_{0}\alpha \left(D_{t}+D_{g}\right)}$$

where h is the Planck's constant.

The quality factor is function of the OSNR, the optical bandwidth $B_{0}$ and the electrical bandwidth of the receiver filter $B_{c}$ as given by [14].

(9) $$Q=\frac{OSNR}{\sqrt{2OSNR+1}+1}\sqrt{\frac{B_{0}}{B_{c}}}$$

In terms of the bit-error rate, the performance of the chosen apodization profile is found using:

(10) $$BER=0.5erf\left(\frac{Q}{\sqrt{2}}\right)$$

Many apodization functions are used to improve the performance of fibre transmissions using Bragg's gratings for chromatic dispersion compensation [22]. Apodization reduces internal interference effects due to group delay and chirped gratings offer ideal properties for the linear compensation of dispersion in fibres [23]. A Gaussian apodization function is used to evaluate the chromatic dispersion compensation performance of the proposed design for given refractive indices (n = 1.46) and is readily available in *Optisystem 18*. Gaussian apodization function g(z) is given by:

(11) $$g\left(z\right)=exp\left(-a{\left(\frac{z}{l}-0.5\right)}^{2}\right)$$

where *a* is the apodization strength and *x* is the variable grating length.

0≤x≤L1≤a≤25.0<z<l

## 3 Experimental setup in Optisystem 18

In this section, we designed 10 Gb/s and 40 Gb/s models for dispersion compensation using a cascade of four uniform FBG, a Gaussian linear chirped fibre with variable length and optimized for n=1.46 FBG refractive index. The proposed design has been compared to prior works in [2] and [18] using refractive indices n=1.45 and n=1.47 in their investigations of the effects of chromatic dispersion compensation using Bragg's gratings.

**Figure 2:**
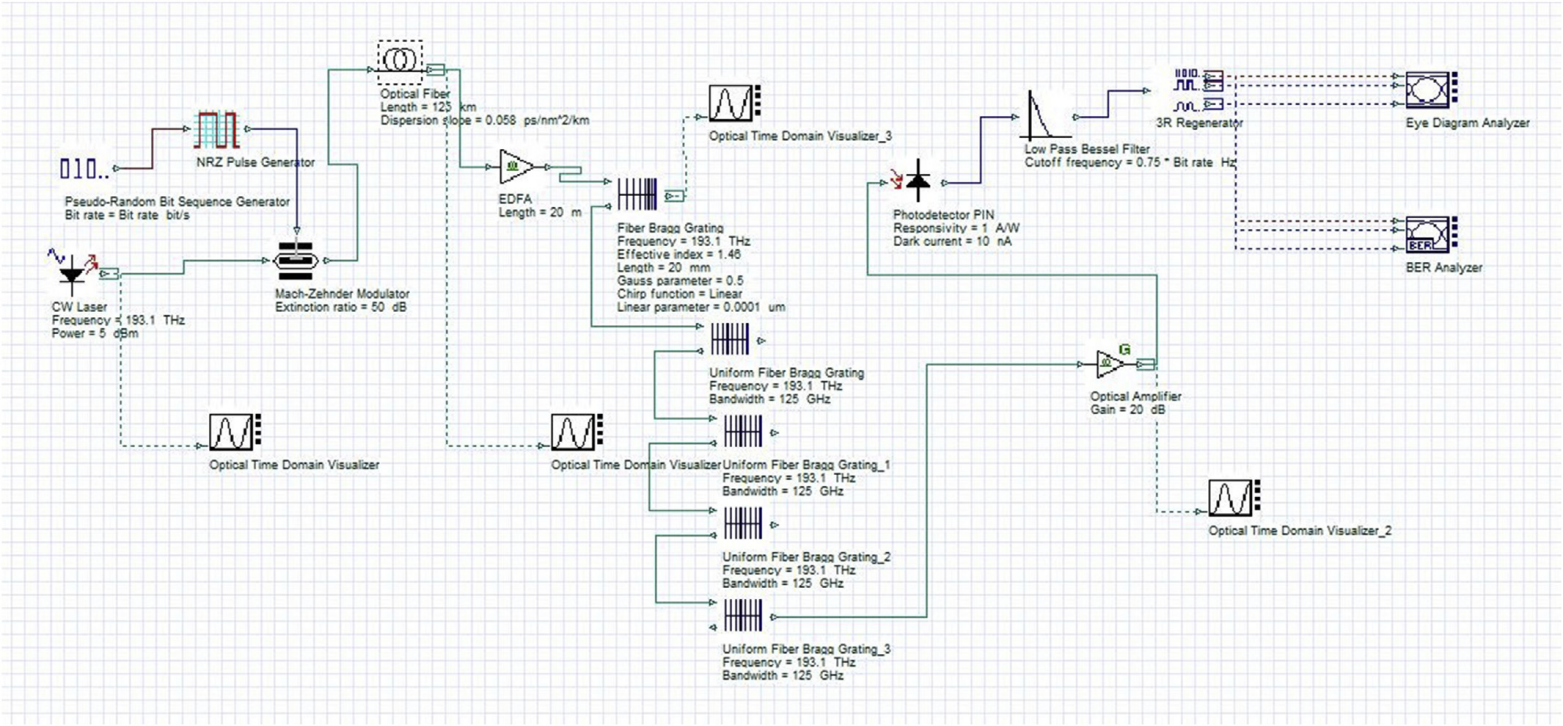
A 10 Gb/s model using a cascade of four-uniform fibre Bragg grating and EDFA.

Apodized cascaded fibre gratings have been used in [21] for post-dispersion compensation with spectral width reduction in WDM networks. In the proposed simulation model as illustrated in Figure 2, the generated optical signal by the transmitter is injected through the fiber and amplified by the EDFA before it reaches the chirped fibre gratings section. The output signal is then filtered by a cascade of four uniform fibre Bragg gratings to enhance dispersion compensation before the light pulse is detected and analyzed at the receiver. The output of the fourth uniform FBG is connected to the optical amplifier with a gain of 20 dB and a noise figure of 4 dB to amplify the received signal before detection. A PIN photodiode is used to detect the optical signal and convert it into an electrical signal. The electrical signal is smoothed by a low pass fourth-order Bessel filter and regenerated using a 3R regenerator component available in Optisystem. The 3R regenerator is used to simplify the proposed simulation model and performs three main operations namely regeneration of amplitude, the signal waveform and synchronization of the received signal. The BER analyzer is used to manifest the BER pattern. The eye-diagram analyzer gives the simulated results in the shape of an eye-diagram and computes the corresponding Q-factor and minimum achievable BER.

Simulation parameters for the transmitter are presented in Table 1.

**Table 1:** Simulation parameters of the transmitter.

Parameters	Value
Input power	5 dBm
Pulse generator	Non return to Zero (NRZ)
Bit rate	10 Gb/s
CW Laser frequency	193.1 THz
Mach–Zender modulator extinction ratio	50 dB

Simulation parameters for the chirped FBG are presented in Table 2.

**Table 2:** Simulation parameters of the chirped FBG.

Parameters	Value
Apodization function	Gaussian
Chirp function	Linear with linear parameter 0.0001μm
Grating length	20 mm
Effective core refractive index used, $n_{eff}$	1.46
Gaussian parameter	0.5

The parameters of the transmission channel and receiver used for simulation are presented in Tables 3 and 4.

**Table 3:** Simulation parameters of the optical fibre transmission channel.

Parameters	Value
Optical fibre cable length	variable
EDFA length	20 m
Dispersion	17 kps/nm/km
Dispersion slope	0.058 ps/nm^2^/km
Attenuation	0.2 dB/km

**Table 4:** Simulation parameters of the receiver.

Parameters	Value
Optical amplifier	20 dB
PIN photodiode	Responsivity = 1A/W
	Dark current = 10 nA
Bessel filter	Cut-off frequency = 0.75×Bit rate

**Figure 3:**
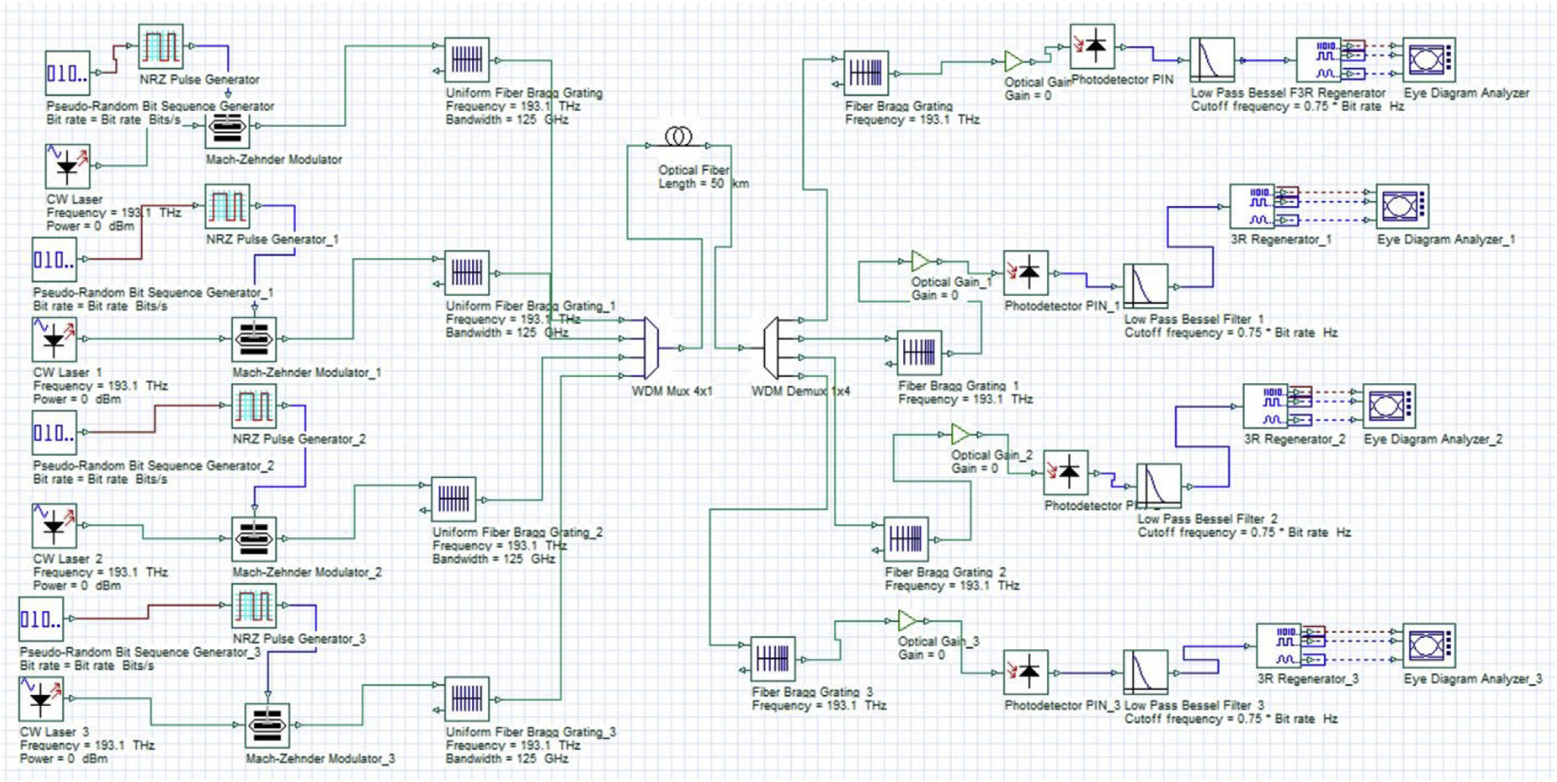
A 40 Gb/s four-channel model using fibre Bragg grating for chromatic dispersion compensation.

Figure 3 presents the simulation model of chromatic dispersion compensation using fibre Bragg gratings for a n = 1.46 linear Gaussian apodized four-channel fibre link. Before transmission, the injected light pulses are individually filtered through a uniform fibre Bragg grating element. A 4 × 1 multiplexer is used to combine the four individual pulses with respective frequencies 193.1 THz, 193.3 THz, 193.5 THz and 193.7 THz and injected through the fibre link. A 1 × 4 demultiplexer element is used to detect and select the received pulses. Before detection by the PIN photodetector, the light pulses are amplified by an EDFA element and compensated for chromatic dispersion using the chirped FBG element. An Eye-diagram analyzer element is connected to the 3R regenerator modules and records the quality factor of the received signal and its corresponding Eye-diagrams.

## 4 Results and discussion

**Figure 4:**
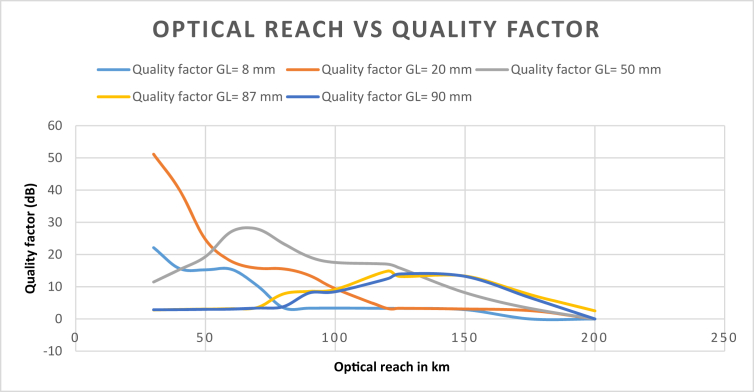
Optical reach versus Quality comparison graph for different grating lengths.

Quality factor results for linear Gaussian apodization for the proposed model were obtained respectively for different grating lengths as recorded in Table 5 considering a SMF link with variable optical reach. The corresponding bit-error rates are also recorded for each case. A comparative diagram of the recorded quality factors against the SMF optical reach is plotted in Figure 4.

**Table 5:** Optical reach versus Quality factor for different chirped grating lengths.

Optical reach in km	Quality factor GL = 8 mm	Quality factor GL = 20 mm	Quality factor GL = 50 mm	Quality factor GL = 87 mm	Quality factor GL = 90 mm
30	22.13	51.14	11.48	2.80	2.87
40	15.64	40.04	15.34	2.95	2.89
50	15.26	24.63	19.36	3.07	2.99
60	15.41	17.91	27.14	3.19	3.06
70	10.29	15.77	27.92	3.56	3.39
80	3.48	15.56	23.41	7.79	3.83
90	3.35	13.52	19.28	8.56	8.09
100	3.39	9.42	17.53	9.16	8.47
120	3.29	3.28	17.02	14.85	12.46
125	3.28	3.32	15.77	13.19	13.97
150	2.89	3.09	8.14	13.33	13.23
175	0	2.60	3.29	7.58	6.62

For a 10 Gb/s link, the effects of chromatic dispersion are greatly reduced using a hybrid design of apodized fibre Bragg grating, and uniform Bragg grating. Table 5 presents the Q-factor results of the fibre length corresponding to each grating length used. The Q-factor results for a grating length of 20 mm are more consistent and give a significant result of 51.14 dBm at 30 km. A Q-factor of 51.14 dBm obtained for 30 km using our simulation model for a link with a refractive index of n=1.46 is significantly higher than the quality factor results obtained at 30 km for 8 mm, 50 mm, 87 mm, and 90 mm of fixed chirped grating length.

**Figure 5:**
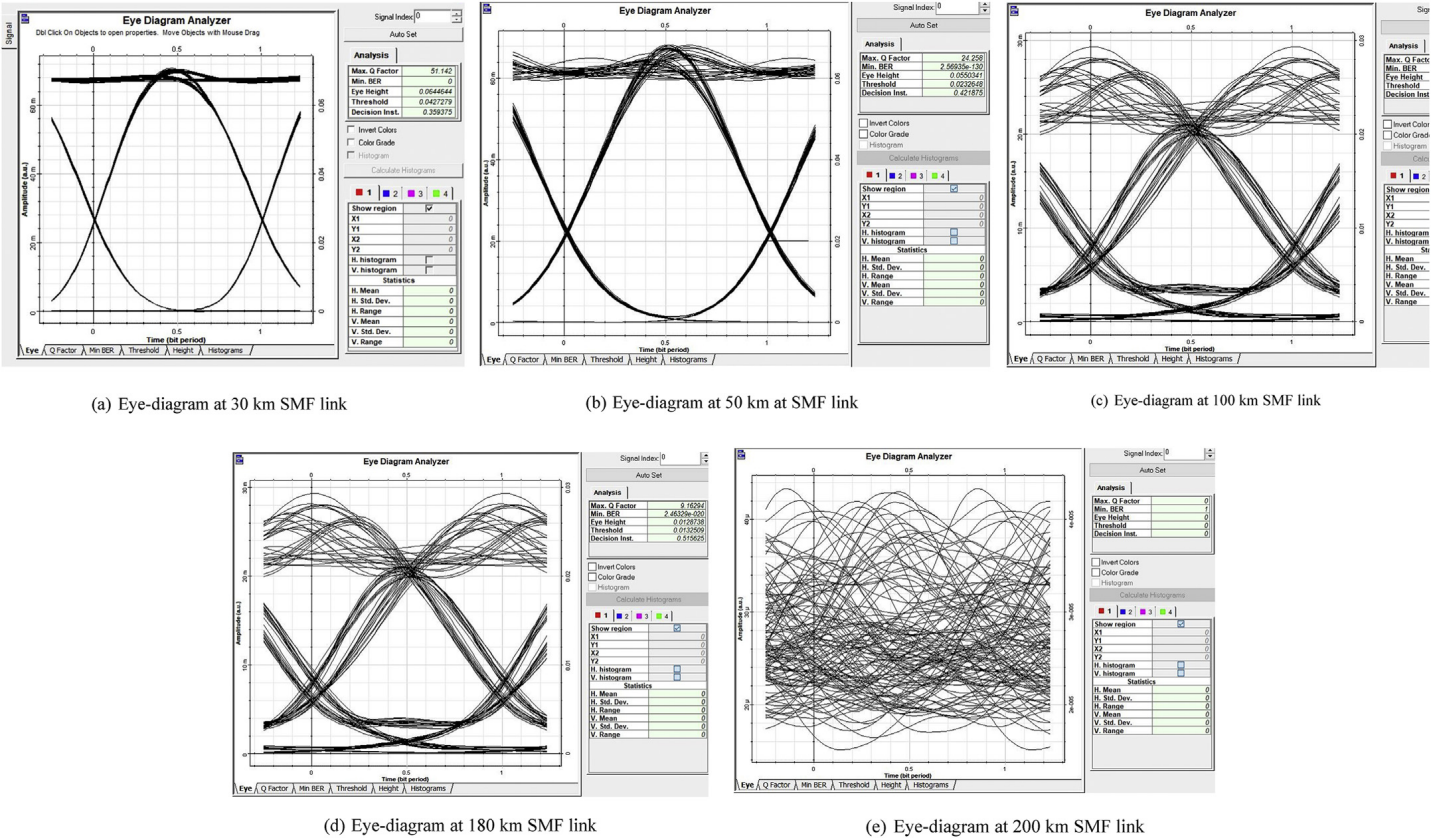
Eye-diagrams for different optical lengths for a 10 Gb/s model.

**Figure 6:**
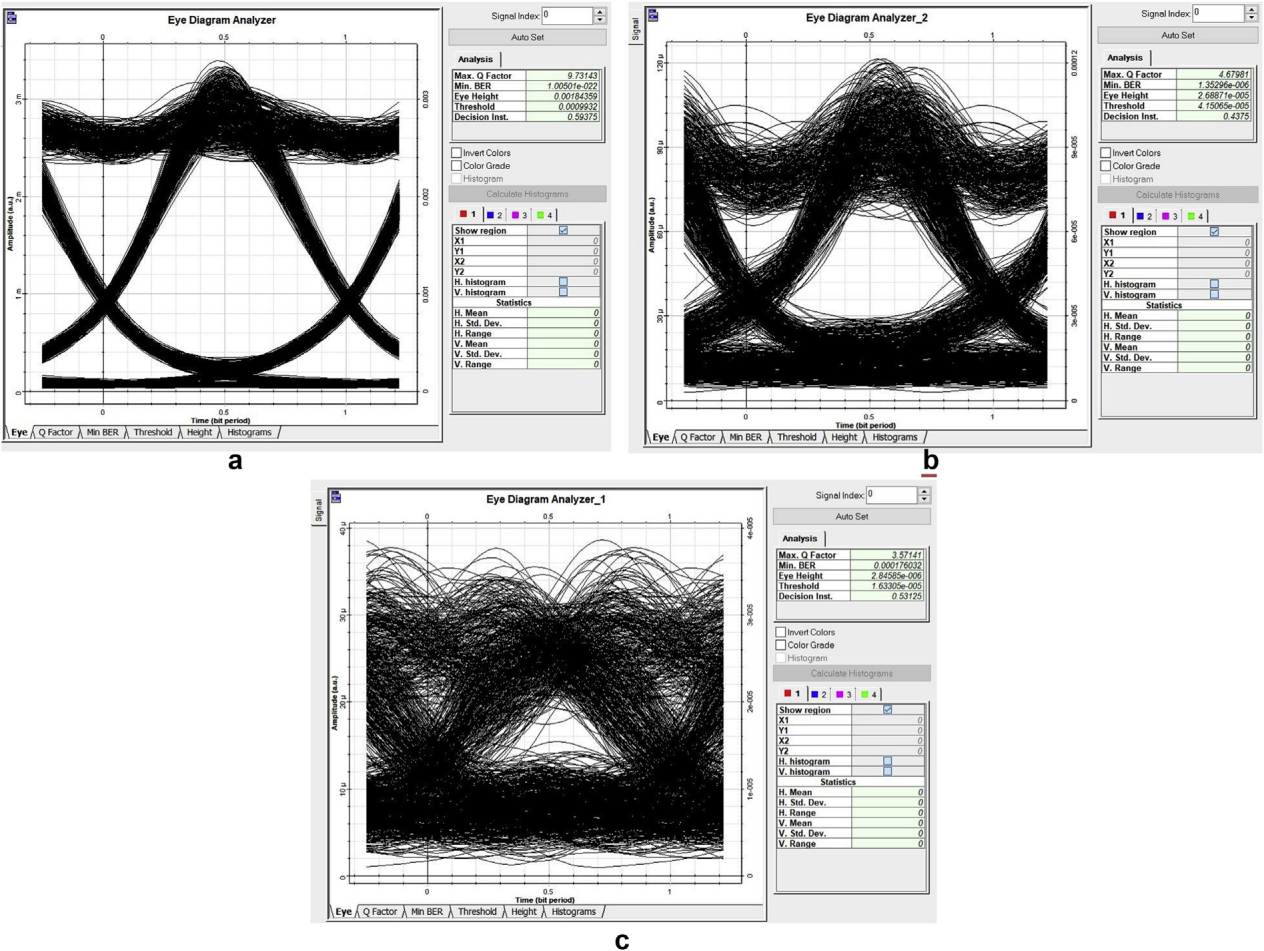
Eye-diagrams for 40 Gb/s link using 20 mm CFBG.

The Q-factor results obtained for 40 Gb/s four-channel link agree with theoretical predictions in on the increasing effects of chromatic dispersion from 10 Gb/s to 40 Gb/s optical transmissions [3]. For varying grating lengths and variable SMF optical length, the Q-factor also varies as given in Figure 5. At 30 km, and for a grating length of 20 mm; the achievable maximal Q-factor and eye-diagram quality in 40 Gb/s link is reduced at least three times as it can be seen in Figure 6. As the optical reach distance increases, chromatic dispersion is experienced differently depending on the data bit rate.

A comparison graph of the Q-factor results is given in Figure 4. The Q-factor results for grating length of 20 mm consistently decrease as the SMF length increases. For other grating lengths, the Q-factor results fluctuate before decreasing over increasing SMF length.

The performance of the proposed simulation model for 10 Gb/s is also illustrated using the eye-diagrams for different SMF lengths and corresponding chirped grating segments. Figure 5 gives eye-diagrams using different optical lengths for a chirped Bragg grating length of 20 mm at 30 km, 50 km, 100 km, 180 km and 200 km. A clear eye-diagram is obtained at 30 km and the eye-diagram blurs as the SMF optical length increases. A null Q-factor result obtained respectively for 8 mm, 20 mm, 50 mm and 90 mm of chirped grating length at 200 km SMF make the use of the proposed design unsuitable for optical communication.

In Figure 6, the effects of chromatic dispersion for a 40 Gb/s link gave a fifth of the quality factor obtained for a 10 Gb/s link for 20 mm grating length and 30 km SMF length. For 20 mm chirped grating length, no signal is detectable at the receiver for a 40 Gb/s four-channel link past the 60 km mark as shown in Figure 6 (c). As the SMF distance increases from 30 km to 40 km, the Q-factor result consistently decreases as shown in Figure 6 (b).

A comparison of Q-factor results for 10 Gb/s link for a FBG refractive index of n=1.45, n=1.46 , and n=1.47 is given in Table 6. Theoretically, satisfactory standards of Q-factor and BER are respectively Q > 6 and BER ≤10^−9^ [18]. The results show that for n=1.46 refractive index, a significant Q-factor result is obtained for 45 mm grating length using the proposed design at 150 km than that obtained in [18]. At 87 mm, results obtained using the proposed design are slightly above the threshold theoretical satisfactory Q-factor value and is less than that obtained in [2]. In each case however, obtained BER results are less than the minimum BER value of 10^−9^. The current technology using FBG designs to mitigate effects of linear dispersion is less costly than implementing optical communication systems that rely on coherent optical systems and digital signal processing. However, the revived interest in coherent optical systems is due to the need to increase spectral efficiency and data rates in fibre links [24].

**Table 6:** Quality Factor Comparison with previous work using chirped fibre grating.

References	FBG Refractive index	SMF length in km	CFBG length in mm	Quality factor in dBm	BER
[2]	1.45	210	87	23.14	7.04 × 10^−11^
[18]	1.47	150	45	12.74	1.63× 10^−37^
This work	1.46	150	45	15.64	1.96× 10^−55^
This work	1.46	175	87	7.58	1.62× 10^−14^

## 5 Conclusion

This paper presents a comparative analysis of the performance of a hybrid design of linear Gaussian chirped FBG and a cascade of uniform FBG for fibre compensation in 10 Gb/s and 40 Gb/s fibre transmission link using different chirped fibre Bragg grating lengths for a refractive index of n=1.46.

The designs were implemented using *OptiSystem 18* simulator for long distance optical communication. For constant FBG effective refractive index (n = 1.46), different Q-factor results are determined for different fibre lengths by varying the grating length. The performance is calculated for each apodization profile with reference to Q-factor, eye-diagram and BER. It was found that by applying the proposed design for a 10 Gb/s link, the achievable maximum Quality factor is 51.14 dBm at 30 km using a chirped fibre length of 20 mm and 9.73 dBm for a four-channel 40 Gb/s link. At 150 km, obtained Q-factor and minimum BER results for a FBG index of 1.46 are respectively 15.64 dBm and 1.96× 10^−55^. The results found for a 10 Gb/s design using a cascade of four uniform FBG and chirped FBG and for a 40 Gb/s model agree with theoretical predictions with regard to the effects of chromatic dispersion in long communication optical links. The Q-factor results obtained using the proposed simulation model offer significant improvement compared to previous works.

## Declarations

### Author contribution statement

Isidore Nsengiyumva: Conceived and designed the experiments; Performed the experiments; Analyzed and interpreted the data; Contributed reagents, materials, analysis tools or data; Wrote the paper.

Elijah Mwangi, George Kamucha: Analyzed and interpreted the data; Contributed reagents, materials, analysis tools or data; Wrote the paper.

### Funding statement

This work was supported by the African Union through the Pan African University scholarship scheme.

### Data availability statement

Data included in article/supplementary material/referenced in article.

### Declaration of interests statement

The authors declare no conflict of interest.

### Additional information

No additional information is available for this paper.
